# Effects of *Zataria Multi-Flora*, Shirazi thyme, on the Severity of Premenstrual Syndrome

**DOI:** 10.5812/nms.13709

**Published:** 2013-12-09

**Authors:** Marzieh Sodouri, Negin Masoudi Alavi, Nahid Fathizadeh, Mohsen Taghizadeh, Zohreh Azarbad, Mohammadreza Memarzadeh

**Affiliations:** 1 Trauma Nursing Research Center, Kashan University of Medical Sciences, Kashan, IR Iran; 2 Nursing and Midwifery Care Research Center, School of Nursing and Midwifery, Isfahan University of Medical Sciences, Isfahan, IR Iran; 3 Department of Nutrition, Kashan University of Medical Sciences, Kashan, IR Iran; 4 Department of Society Medicine, Kashan University of Medical Sciences, Kashan, IR Iran; 5 Research Center of Barij Essence, Kashan, IR Iran

**Keywords:** Premenstrual Syndrome, Clinical Trial, Herbal Medicine

## Abstract

**Background:**

Premenstrual Syndrome (PMS) is a common problem in women. *Zataria multiflora Boiss* is a valuable plant. This herbal plant has been used in different conditions.

**Objectives:**

The study was performed to investigate the effects of *Zataria multiflora Boiss* to control PMS symptoms.

**Materials and Methods:**

This study was a double-blinded, prospective randomized trial. The study population was the college students living in the dormitories of Isfahan University. Eighty-eight eligible students were randomly divided to intervention and control groups. Students completed the prospective record of the impact and severity of menstrual symptoms (PRISM) calendar including 30 PMS symptoms for four menstrual cycles (two cycles before, and two after the intervention). The intervention group received pearls containing 20 mg of *Zataria multi-flora* essence (four pearls each day) for two menstrual cycles, seven days before menstruation. The mean difference of PMS severity score between groups was analyzed with Mann-Whitney U test. The difference in frequency score of symptoms was analyzed using repeated-measure analysis of variance.

**Results:**

Thirty-eight students in intervention and 37 students in placebo group completed the study. The groups had no significant difference regarding the severity of PMS. Both groups showed a significant improvement in PMS severity score after the intervention. The repeated-measure analysis of variance showed that the frequency of symptoms decreased significantly in the cycles, but it was not different in intervention and placebo groups.

**Conclusions:**

Our findings did not support the use of *Zataria multiflora Boiss* in premenstrual syndrome.

## 1. Background

Premenstrual Syndrome (PMS) is a common problem in women (1). This syndrome was first described in 1931 by Frank and Horney (2). PMS is a term refers to a series of psychological and physical symptoms which some women experience in the late luteal phase (7-14 days before menstruation) of their menstrual cycle (3). It is estimated that 85-90 % of women have PMS before menopause (4). Premenstrual dysphoric disorder is a severe form of PMS (5), with a prevalence of 3-8% (6), and severe psychological conditions such as reduced mental health, and mood disorders, especially depression (5). In Iran the prevalence of PMS is 67-78.4% (7, 8). PMS causes approximately three billion pounds lost annually in the Britain, due to reduced work efficiency (9). PMS etiology is unknown. Fluctuations in estrogen and progesterone, genetic and neurobiological factors have been cited as contributing factors (6). Diet and lifestyle are also important in creating this syndrome. Given the uncertainties associated with the causes of this syndrome, there are also no universal acceptable treatments for this problem (5, 6, 10). About two hundred different symptoms have been listed for PMS. Mild to moderate PMS symptoms can be relieved by changes in lifestyle, but most severe symptoms require pharmacological interventions (3). A systematic review in Iran showed that the wide range of pharmacological and non-pharmacological interventions such as diuretics, gonadotropins, progesterone, and supplements (vitamins and minerals), exercise, massage, yoga, phototherapy, dietary changes and herbal remedies have been suggested for controlling this syndrome (10). Pharmacological interventions are expensive; with side effects and their effectiveness are under question (4, 9, 10). Patients with this disorder tend to use complementary therapies. The results of a telephone survey in America showed that approximately 80% of patients with PMS use complementary therapies (11). The use of herbal medicine as a new source of prevention and treatment of different problems has become prevalent worldwide (12, 13). Herbal products such as Hypericum (14), Saffron (11), Vitex agnus- castus extract (15), and Ginkgo (16) have been used to treat PMS. Zataria multiflora Boiss (ZM) is a thyme-like plant belonging to the Labiatae family which grows wild in Iran, Pakistan and Afghanistan. This plant with the vernacular name of "Avishan-e-Shirazi" (Shirazi thyme) in Iran is a valuable medicinal and condimental plant (17). ZM is a perennial plant with a woody, fibrous root, and its leaves are small, narrow, elliptical, and greenish grey (18). Plants of Labiatae family have been used in traditional medicine for exhaustion, weakness, depression, memory enhancement, circulation improvement, strengthening of fragile blood vessels, inflammation, infection, indigestion, and gastritis (19). The extract of this plant has been used to treat coughs, bronchitis, laryngitis, and the common cold. ZM is used as an antibacterial agent in oral hygiene by traditional healers in Iran (18). This herbal plant has been used as an antidyspepsia, and antiseptic (20). Researches show that compounds containing ZM, were able to inhibit mediators of inflammatory reactions (14), and act as antioxidant (21, 22), and antispasmodic (23, 24), with analgesic effects (25). In a research, ZM was effective in the treatment of primary dysmenorrhea (26). ZM contains organic oils of thymol and carvacrol with antispasmatic, and anti-inflammatory effects (13, 24). These effects might help to control the symptoms of PMS which has not been investigated yet. 

## 2. Objectives

This trial has been designed to investigate the effects of ZM to control PMS symptoms in Isfahani college students. 

## 3. Materials and Methods

This double-blinded, prospective randomized trial study was performed between October 2012 and April 2013. 

### 3.1. Study Sample

**Figure 1.**
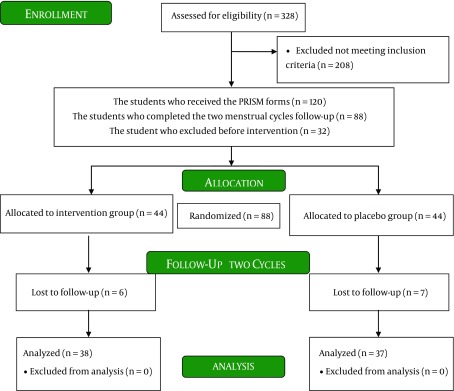
The Consort Flow Chart Describing the Progress of Subjects Through the Study

The study population was college students living in the female dormitories of Isfahan University. The numbers of rooms were selected randomly, and all the students in selected rooms were evaluated for PMS. The sample size was determined with the assumptions: standard deviation and the difference in the mean of severity of PMS symptoms are six and four respectively according to the previous clinical trial in PMS using the same assessment tool (27), 95% confidence level and power of 80%. Forty eight subjects were estimated to be needed in every group. The inclusion criteria for the study were: PMS diagnosis according to the American Psychiatric Association (APA) criteria (27), age between 18 and 35 years, regular menstrual cycles, no history of stressful events in the previous three months (death of relatives, divorce, or hospitalization), and no history of known psychological problems or other chronic diseases. Students who failed to follow the interventions or those who used oral contraceptive pearls or hospitalized during the study period were excluded from the research. In total, 328 students were screened, and 88 students had more than five symptoms in seven days before menstruation which were recruited randomly to the study groups. Thirty eight students in intervention and 37 students in placebo group finished the study. The study framework is presented in Figure 1. 

#### 3.1.1. Instruments

First PMS was screened with a tool containing 13 symptoms designed by Fathizadeh et al. (27). The reliability of the tool was calculated as 0.86. The diagnosis of PMS was made according to the existence of at least five symptoms, including one affective symptom during the last week of the luteal phase for at least two cycles, and the symptoms that interfere with the daily life (27). The eligible students received the prospective record of the impact and severity of menstrual symptoms (PRISM) calendar contained 30 symptoms of the PMS. The symptoms such as irritability, tendency to crying, restlessness, nausea, breast tenderness, abdominal bloating, aggressiveness, insomnia, and appetite change were recorded every day using four scores (0 = none, 1 = mild, 2 = moderate and 3 = sever enough to interferers with the daily life). This tool is a valid test with the reliability of 92% which has been used in previous studies (27). The menstruation was recorded in the calendar. The students completed the PRISM calendar for four menstrual cycles, two before , and two after the intervention. The age, the age at menarche, menses duration, BMI, marriage status, and the educational level were also recorded. 

#### 3.1.2. Intervention

In this study, the medication was prepared as a pearl containing 20 mg of Zataria multi-flora essence or placebo, manufactured by Barij Essence Pharmaceutical Company, Kashan, Iran. All pearls were similar in appearance, size and color. The pearls were coded in the company. Neither student, nor the researchers were aware of the type of drug till the end of the study. For investigating the effects of time as a possible confounding variable, the students were asked to complete the PRISM calendar for two menstrual cycles before, and two cycles after the intervention. After two menstrual cycles, the students were randomly divided to intervention and placebo groups. Each student received 60 pearls. Intervention group received 80 mg of Zataria multi-flora essence (two pearls every 12 hours), seven days before menstruation for two menstrual cycles. The placebo group also received the same amount of pearls containing nonactive ingredients. Treatment compliance was assessed by counting the unused medication and telephone follow up. Students, who had taken at least 75% of the pearls, were considered as compliant. 

### 3.2. Ethical Considerations

The study was performed according to the Helsinki declaration protocol. The objectives of the study were explained to the students, and informed consent was obtained from all participants. Students could leave the study at any time. The study was approved by the Ethical Committee of Kashan University of Medical Sciences.

### 3.3. Data Analysis

The normality of variables was analyzed by Kolmogorov-Smirnov test. The difference of marriage status and educational levels in the groups was analyzed with chi-square test. The Mann-Whitney U test was used for comparing the age, the age at menarche, menses duration, and BMI in the groups. Two outcome variables were determined in this study. The severity of PMS score was calculated with the mean of severity of symptoms in seven days before menstruation. The possible score was between 0 and 90. The mean of PMS severity scores in the two menstrual cycles before and two cycles after the intervention was measured, and its difference in groups was analyzed with Mann-Whitney U test. The pre and post difference in each group was analyzed using Wilcoxon test. The second variable was the frequency of PMS symptom score in seven days before menstruation in every menstrual cycle. The frequency of PMS symptom score in every menstrual cycle could be between 0 and 210 (30 symptoms in seven days). The differences in frequency of PMS symptom score were analyzed with repeated-measure analysis of variance. 

## 4. Results

**Figure 2.**
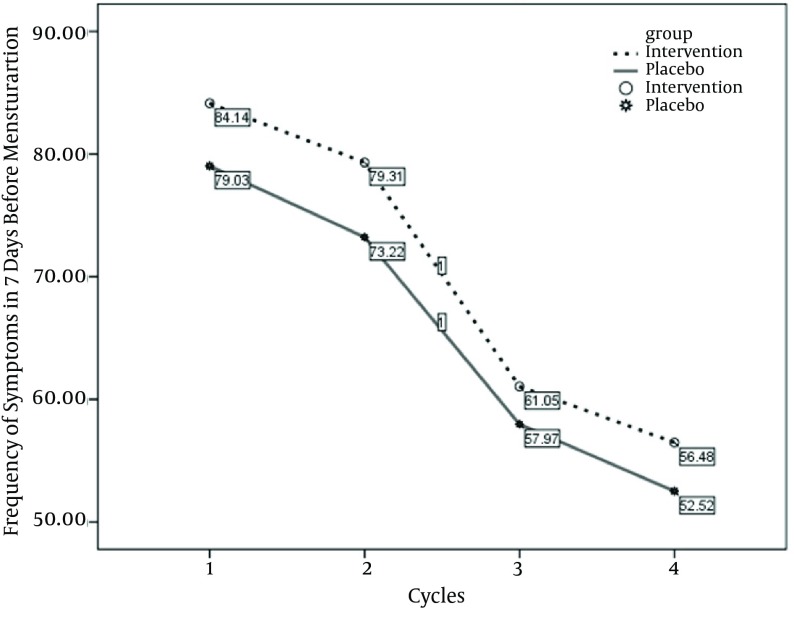
The Frequency of Symptoms in the Menstrual Cycles Before and After the Intervention

The mean age of our study population in intervention and placebo groups were 20.97 ± 2.07 and 22.16 ± 2.63 years, respectively (P = 0.08). Mean BMI was 21.8 ± 2.8 kg/m ^2 ^in intervention, and 20.88 ± 1.98 kg/m ^2 ^in placebo groups (P = 0.247). The students' characteristics including the marriage status, the studying degree, the age at menarche, and menses duration were not significantly different in the groups (Table 1). The mean of PMS severity score was 28.82 in intervention, and 27.72 in control groups before the study, which was not significantly different (P = 0.417). After intervention the mean of PMS severity reduced to 21.76 in intervention and 18.9 in placebo group (P =0.356). Table 2 shows that the mean reduction in severity of PMS score was 6.79 in intervention, and 8.82 in placebo group which did not show any significant difference between the groups (P = 0.157). Although Wilcoxon test showed that the severity of PMS score reduced significantly in both groups after the intervention. (In intervention group: Z = 3.58, P = 0.0001; in placebo group: Z = 4.2, P = 0.0001).The frequency of reported symptom score decreased from 84.13 in the first menstrual cycle to 56.5 in the fourth cycle in intervention group. In placebo group the decrease was from 79 to 52.5. The repeated-measure analysis of variance showed that the frequency of symptom score decreased significantly in the menstrual cycles, but this decrease was not significantly different in intervention and placebo groups (Table 3, Figure 2). 

**Table 1.** The Variables in Intervention and Placebo Groups

Variable	Intervention	Placebo	Value ^c^	P value
**Age, Mean (SD), y** ^**a**^	20.97 (2.07)	22.16 (2.63)	-1.75	0.08
**Age at menarche, Mean (SD), y** ^**a**^	13.26 (1.03)	13.4 (1.59)	-0.33	0.74
**Menses duration, Mean (SD), d** ^**a**^	6.63 (1.34)	6.4 (1.06)	-0.6	0.55
**Body Mass , Mean (SD), Kg/m** ^**2 **^	21.8 (2.81)	20.88 (1.98)	-1.16	0.25
**Marriage, No. (%) ** ^**b**^			0.72	0.52
Single	34 (89.5)	34 (91.9)		
Married	4 (10.5)	3 (8.1)		
**Education No. (%) ** ^**b**^			1.55	0.46
License	16 (42.1)	14 (37.8)		
Master Degree	5 (13.2)	9 (24.3)		
Doctorate	17 (44.7)	14 (37.8)		

^a^ The differences of the means were analyzed by Mann-Whitney U test and the value represents the Z.

^b^ The differences of frequencies were analyzed by chi-square test.

^c^ The value represents Z value in quantitative variables and Chi-square in categorical variables.

**Table 2.** The Premenstrual Syndrome Severity of Symptoms in Intervention and Placebo Groups

The Premenstrual syndrome severity of symptoms	Intervention, Mean (SD)	Placebo, Mean (SD)	Z	P value
**Before intervention**	28.82 (12.28)	27.72 (11.98)	-0.81	0.42
**After intervention**	21.76 (13.28)	18.90 (11.16)	-0.92	0.36
**The difference**	6.79 (8.8)	8.82 (7.26)	-1.42	0.16

**Table 3.** The Frequency of Symptom Score in Different Cycles, and the Effects of Interventional Groups and Cycles Using Repeated-Measure Analysis of Variance

	Cycle 1	Cycle 2	Cycle 3	Cycle 4
**The frequency of symptom score, mean (SD)**				
Intervention	84.13 (40.6)	79.3 (51.1)	61 (47.6)	56.5 (43.2)
Placebo	79 (34.3)	73.2 (41.8)	58 (46.2)	52.5 (42.6)
**The cycles**				
F	8.61			
P value			0.0001	
**Frequency of total symptom score in cycles × Interventional group**				
F	1.13			
P value			0. 35	

## 5. Discussion

The primary goal of this study was to investigate the potential effect of ZM to control the symptoms of PMS. The results showed that the pearls containing Zataria multi-flora essence could not decrease the PMS severity and the frequency of symptoms. Different studies had shown the relieving properties of ZM in different conditions (21-25). In an in-vitro study, Fatemi et al. had reported the antioxidant effects of this herbal plant (28). Majlessi et al. studied the beneficial effects of ZM essential oil (50, 100, or 200 μL/kg) on cognitive function and mental abilities in a rat model of Alzheimer disease. He concluded that ZM may be a potentially valuable source of natural therapeutic agent for the treatment of Alzheimer disease in rat model (29). The therapeutic effect of ZM in respiratory disorders, as an antitussive and its relaxant effect on tracheal smooth muscle of Guinea pig were also documented (18). Moreover, ZM extracted essential oils exhibited significant bacteriostatic and bactericidal activities against Gram-positive and Gram-negative bacteria at concentrations ranging from 0.12 to 8 μL/mL (30). Although the current study, did not show any beneficial effects of this herbal plant in the treatment of PMS. Most of the studies about the ZM effects are in animal models or in-vitro studies. Maybe the wide recommendations of this herbal medicine in different conditions need more clinical investigations. Both placebo and intervention groups showed significant improvement in the severity and frequency of symptoms. Researchers have recommended many pharmaceutical treatments for PMS such as: Magnesium and vitamin B6 (27), vitamin D (31), Vitex agnus castus extract (32).While many drugs for PMS have been evaluated, all have significant limitations and none of them has reported the efficiency greater than 60 - 70% (31). Our study showed that the same effects can be observed following the use of placebo. Previous data in the literature showed that over 20% of patients submitted to placebo treatment in PMS studies had a major improvement in their symptoms (33). In the study performed by De Souza et al. placebo led to a significant decrease in all the PMS symptoms (34). In the studies performed on PMS, the placebo effect on the syndrome was considerable. It seems that paying attention to the women under study can bring about a positive psychological effect on PMS treatment (27). In our study the placebo response was greater than expected. A placebo contains no ingredient active against the target condition; therefore, it can have no activity. However, when placebos are used in randomized clinical trials they produce an effect larger than what observed in no treatment control groups. The current concepts of the ‘placebo response’ come from trials in which dummy treatments have been used as the ‘control’ for the active treatments. If we are certain that a drug is effective, then we need to make sure that it works better than giving ‘nothing’. However, the response to the dummy treatment cannot be a response to the ‘nothing’ that is in the tablet, it must be a response to ‘something’. That something could be the many ‘contextual factors’ or ‘incidental effects’ surrounding the administration of an intervention (35). Our study showed that PMS might be a problem which contextual factors can increase the placebo response. The studies in evaluating the treatments of PMS without the use of control group and placebo are under serious questions. Even in randomized clinical trials, there must be some policies in reducing the placebo responses. Follow-up of women for more than three menstrual cycles can limit the placebo responses in PMS interventional studies (30). In the current research the length of treatment (two menstrual cycles) might not be sufficient to minimize the placebo effect. In conclusion, PMS is a common and disturbing health condition which can have many individual and social problems for women and their families. This condition can cause family conflicts, and decrease the efficacy of women. Unfortunately the current treatments for this common condition are not satisfactory and many women seek alternative and complementary medicine including herbal remedies for reliving the symptoms of PMS. We assumed that ZM with its wide range of therapeutic usage might have positive effects in controlling the severity of PMS. Our findings did not support the use of ZM in premenstrual syndrome. It might be that the dosage used in this study could not control the symptoms of PMS. The effects of other dosages need further investigation. This trial had some limitations. The women were received treatment for seven days in every menstrual cycle and for two cycles. The length of treatment might not be sufficient in controlling the symptoms of PMS. Recording 30 symptoms every day for four menstrual cycles was difficult for students, and many did not complete the study. We recommend the use of brief form of the PRISM calendar in future studies. 
